# Clinical Performance of Rapid Antigen Tests for the Detection of SARS-CoV-2 Infection in the Emergency Department and Community: A Retrospective Study

**DOI:** 10.1155/2022/9447251

**Published:** 2022-10-06

**Authors:** Pei-Chin Lin, Hung-Pin Chiu, Fang-Yi Cheng, Chih-Chun Chang, Fang-Yeh Chu

**Affiliations:** ^1^Department of Clinical Pathology, Far Eastern Memorial Hospital, New Taipei City, Taiwan; ^2^Department of Nursing, Cardinal Tien Junior College of Healthcare and Management, Yilan, Taiwan; ^3^Graduate School of Biotechnology and Bioengineering, Yuan Ze University, Taoyuan City, Taiwan; ^4^Department of Medical Laboratory Science and Biotechnology, Yuanpei University of Medical Technology, Hsinchu City, Taiwan; ^5^School of Medical Laboratory Science and Biotechnology, Taipei Medical University, Taipei City, Taiwan

## Abstract

**Background:**

Rapid antigen tests for severe acute respiratory syndrome coronavirus 2 (SARS-CoV-2) detection have been authorized for emergency use (EUA); however, the performance has not been fully evaluated in clinical contexts. This study aimed to provide evidence regarding the diagnostic performance of SARS-CoV-2 rapid antigen tests compared with the real-time reverse transcription-polymerase chain reaction (RT-PCR) test in the emergency department (ED) and community.

**Methods:**

Patients who underwent SARS-CoV-2 rapid antigen tests using the VTRUST COVID-19 Antigen Rapid Test (TD-4531) and real-time RT-PCR on the same day in the ED or community from May 24, 2021, to June 24, 2021, were examined.

**Results:**

Paired nasopharyngeal swabs were collected from 4022 suspected COVID-19 patients: 800 in the ED and 3222 in the community. Overall, 62 (1.54%) tested positive, 13 tested indeterminate, and 3947 tested negative by real-time RT-PCR. The sensitivity and specificity of the antigen test were 51.61% and 99.44% (overall), 62.50% and 99.61% (ED), and 31.82% and 99.40% (community), respectively. There were 30 false negatives and 22 false positives. Among the false negatives, 16.67% had a cycle threshold (Ct) value of <25.

**Conclusion:**

The VTRUST COVID-19 Antigen Rapid Test showed comparable specificity as real-time RT-PCR for the ED and community, but the sensitivity was relatively low, especially when the Ct value was >25. This test can be useful for the rapid identification of infected subjects in an epidemic situation.

## 1. Introduction

Since December 2019, severe acute respiratory syndrome coronavirus 2 (SARS-CoV-2) has spread around the world in a short period of time. The World Health Organization (WHO) declared coronavirus disease 2019 (COVID-19) a Public Health Emergency of International Concern on January 30, 2020 [1]. An outbreak of COVID-19 occurred in May 2021 in Taiwan [2, 3]. There is an urgent need for a prompt and accurate diagnostic tool for SARS-CoV-2 infection.

Tests for SARS-CoV-2 can be used for diagnosis, which is crucial as infection control measures to limit onward spread [4]. Real-time reverse transcription-polymerase chain reaction (RT-PCR) is considered the reference standard for SARS-CoV-2 detection. However, RT-PCR testing requires trained personnel and is relatively time-consuming and expensive [5]. During the pandemic, the test capacity of RT-PCR was unable to meet the demands. In contrast, SARS-CoV-2 rapid antigen tests are cheaper, with a rapid turnaround time and less complexity [6]. In order to expand test capacity, many SARS-CoV-2 rapid antigen tests have been authorized for emergency use (EUA) and are widely used in the hospital setting, community, and even at home [7]. However, the sensitivity for SARS-CoV-2 detection of these rapid tests is lower than that of RT-PCR [6]. There have been concerns about the clinical performance of SARS-CoV-2 rapid antigen tests due to inadequate data [8].

Although some studies described the diagnostic performance of different SARS-CoV-2 rapid antigen tests in the emergency department (ED) and community, the diagnostic performance of antigen tests varied [9–11]. Similarly, some new rapid antigen tests were developed and authorized for emergency use in Taiwan, such as VTRUST COVID-19 Antigen Rapid Test (TD-4531, Taidoc technology corporation, Taiwan) which employs a lateral flow chromatographic immunoassay method with anti-SARS-CoV-2 N protein IgG [12]. The VTRUST COVID-19 Antigen Rapid Test was one of antigen tests that received EUA in Taiwan and was widely used during the outbreak. However, to the best of our knowledge, no studies have reported the performance of the VTRUST COVID-19 Antigen Rapid Test in clinical settings. Therefore, this study aimed to evaluate the clinical performance of VTRUST COVID-19 Antigen Rapid Test (TD-4531) for COVID-19 in the ED and community testing sites.

## 2. Materials and Methods

### 2.1. Subject Selection and Study Design

This retrospective study included subjects who received SARS-CoV-2 rapid antigen tests and real-time RT-PCR tests on the same day in the ED of Far Eastern Memorial Hospital (FEMH) or Sanchong District community testing site, New Taipei City, Taiwan (R.O.C.), from May 24, 2021, to June 24, 2021. Subjects younger than 20 years were excluded from this study. The community testing site provided SARS-CoV-2 tests to those in the community who wanted tests. Paired nasopharyngeal swabs were collected from these subjects for both the SARS-CoV-2 antigen test and real-time PCR test.

Epidemiological and clinical data were obtained via the chart review from electronic medical records, including age, sex, body temperature (BT), respiratory rate (RR), and oxygen saturation (SpO_2_) at the triage of the ED, symptoms (respiratory, gastrointestinal, flu‐like symptoms, and loss of smell and taste), duration of symptoms, travel history, occupation, cluster history and contact history, results of SARS-CoV-2 antigen test, and results of real-time RT-PCR. This study was approved by the Ethics Committee of FEMH (approval date: 2021/08/23 and 2021/08/18 and approval numbers 110158-E and 110162-E). The requirement for written informed consent was waived due to the retrospective nature of the study and the use of deidentified data. All procedures used in this study adhered to the tenets of the Declaration of Helsinki.

### 2.2. Determination of the SARS-CoV-2 Antigen Test

SARS-CoV-2 antigen test was conducted on-site (in the ED or community testing site) using VTRUST COVID-19 Antigen Rapid Test (TD-4531, Taidoc Technology Corporation, Taiwan) according to the manufacturer's instructions [12]. The VTRUST COVID-19 Antigen Rapid Test is a lateral flow chromatographic immunoassay for the detection of nucleocapsid (N) antigen of SARS-CoV-2. The nasopharyngeal swab specimen is immersed into an extraction tube with 10 drops (about 500 *µ*L) of extraction buffer. The swab is rolled, pressed the head against the bottom and side of the extraction tube, and left in the buffer for 30 seconds. Then, the swab is removed and the 3 drops (about 100 *µ*L) of processed sample is added into the sample well of the test cassette. The specimen migrates by capillary action along the test strip. Results were recorded after 15 minutes of reaction time and were interpreted by the operator based on the visual presence or absence of control and test lines. The test was interpreted as positive if both control and test lines were visually present. The test was invalid if the control line was not present.

### 2.3. Determination of Real-Time RT-PCR

The other nasopharyngeal swab sample was mixed in a universal transport medium and transported to the molecular biology laboratory in FEMH for a real-time RT-PCR test within a few hours. The real-time RT-PCR test was conducted by automated Roche Cobas 6800 SARS-CoV-2 test (Roche, Pleasanton, CA, USA) (target *E* gene and ORF-1ab gene) [13]. Samples were identified as positive if two SARS-CoV-2 targets were detected. Samples were identified as indeterminate if only a single SARS-CoV-2 target was detected.

### 2.4. Statistical Analysis

Descriptive statistics were used to describe epidemiological and clinical data. Continuous data were presented as mean with standard deviation (SD) or medians with interquartile range (IQR), as appropriate. Categorical data were presented as numbers with percentages. Real-time RT-PCR results were considered as the reference standard. The sensitivity, specificity, positive predictive value (PPV), and negative predictive value (NPV) of the VTRUST COVID-19 Antigen Rapid Test were calculated. For sensitivity analysis, diagnostic accuracy was measured at cycle threshold (Ct) values of <20, 20 to 24.99, 25 to 30, and >30 for the *E* gene. Data were analyzed using an online statistical tool [14] and IBM SPSS Statistics for Windows (Version 19, IBM Corp., Armonk, NY).

## 3. Results

**Figure 1:**
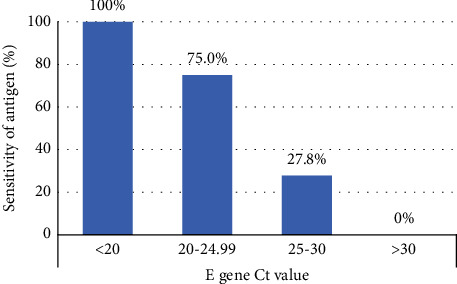
The sensitivity of the VTRUST COVID-19 Antigen Rapid Test with respect to Ct values of RT-PCR for the *E* gene. Ct cycle threshold; RT-PCR, reverse transcription-polymerase chain reaction; COVID-19 coronavirus disease 2019.

During the study period, 4022 paired nasopharyngeal swabs were collected, including 800 from the ED, and 3222 from the community testing site. Overall, there were 62 (1.54%) positive RT-PCR results (median *E* gene Ct value: 24.93; IQR: 21.41–28.79; min-max: 12.88–36.63), as well as 13 indeterminate and 3947 negative RT-PCR results. The sensitivity, specificity, PPV, and NPV of the VTRUST COVID-19 Antigen Rapid Test are presented in Table 1. There were 30 false negatives and 22 false positives. The sensitivity of the VTRUST COVID-19 Antigen Rapid Test was measured with respect to Ct values of RT-PCR for the *E* gene (Figure 1).

The 800 paired samples were collected from 734 patients and 66 caregivers in ED. There were 40 (5.00%) positive RT-PCR results (Table 1). Among 734 patients in ED, 38 patients (5.18%) were tested with a positive RT-PCR result (median *E* gene Ct value: 23.38; IQR: 20.13–26.46; min-max: 12.88–34.2). The characteristics of patients in the ED are shown in Table 2.

Among the symptomatic patients (*n* = 609), 37 (6.08%) had a positive RT-PCR result (median *E* gene Ct value: 23.23, IQR: 20.12–26.84; min-max: 12.88–34.2) and the sensitivity of the antigen test was 62.16%. In 37 patients with positive RT-PCR, the median time from symptom onset to sampling was 3 days (IQR: 1–6 days; min-max: 0–14 days) and 14 patients (37.84%) had a contact history with confirmed cases. In symptomatic patients with negative RT-PCR results, the median time from symptom onset to sampling was one day (IQR: 0–3 days; min-max: 0–60 days) and 10 patients (1.75%) had a contact history with confirmed cases. There were 14 false negatives and no false positives in symptomatic patients in the ED. The median *E* gene Ct value in patients with false negative antigen results was 28.11 (IQR: 23.52–28.46; min-max: 20.11–34.2). The median *E* gene Ct value in patients with true positive antigen results was 21.82 (IQR: 19.01–24.07; min-max: 12.88–27.94). Among the asymptomatic subjects (*n* = 125), only one patient (0.80%) had a positive RT-PCR (*E* gene Ct value: 23.58) and the antigen result was also positive. There were no false negatives and only one false positive antigen result.

The 3222 paired samples were collected from the community testing site. There were 22 subjects (0.68%) with a positive RT-PCR (Table 1). There were 13 subjects with an indeterminate RT-PCR result (only the *E* gene was detected with Ct value > 35). There were 15 false negative and 19 false positive antigen results.

**Figure 2:**
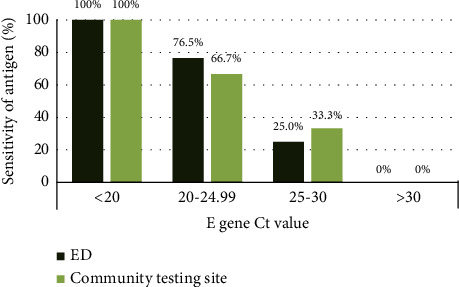
Sensitivity of the VTRUST COVID-19 Antigen Rapid Test with respect to Ct values of RT-PCR for the *E* gene in the ED and community testing site. RT-PCR, reverse transcription-polymerase chain reaction; ED, emergency department; Ct cycle threshold; COVID-19: coronavirus disease 2019.

The sensitivity analysis in the ED and community testing site with respect to Ct values of RT-PCR for the *E* gene is presented in Figure 2.

## 4. Discussion

In our study, the VTRUST COVID-19 Antigen Rapid Test demonstrated comparable specificity (99.44%) and NPV (99.24%), as well as lower sensitivity (51.61%) and PPV (59.26%) compared with that noted for the reference standard real-time RT-PCR in clinical settings, including the ED and community testing site. There was a marked difference in sensitivity between our results and the manufacturer's specifications, which indicated a sensitivity of 93.1% (95% CI: 83.0–97.2%) and specificity of 99.6% (95% CI: 97.7–99.9%) [12]. The similar phenomenon was also observed in a previous study which revealed an inferior sensitivity of antigen tests in the clinical context [15]. This might be due to the wide range of Ct values (median: 24.93; min-max: 12.88 to 36.63) of our clinical specimens, although the Ct value of tested specimens was not provided by the manufacturer's specification. With a Ct value less than 20, 25, or 30, the sensitivity of the VTRUST COVID-19 Antigen Rapid Test was 100%, 84.38%, and 64%, respectively. The sensitivity with a cutoff Ct value less than 25 seemed more comparable to the specification provided by the manufacturer and the requirement for the minimum performance according to the WHO recommendation with a sensitivity of higher or equal to 80% and the specificity of higher or equal to 97% [16].

In our study, there were 15.63% false negatives when the Ct value was lower than 25, which was considered highly contagious. Individuals with a Ct value lower than 30 are infectious [17], even some studies described individuals with a Ct value ≥ 30 may be potentially contagious [18]. Furthermore, patients in an early phase of SARS-CoV-2 infection have low viral loads [19], which would not be detected by the antigen test at first. Therefore, the contagious individuals might be missed if tested only using antigen tests and cause the risk of viral transmission by patients who had a false negative antigen test. It should be watchful that the diagnosis of COVID-19 could not be ruled out with a negative antigen test result, especially in individuals with related symptoms or close contact with confirmed cases.

Most patients in our ED were symptomatic. The sensitivity of the VTRUST COVID-19 Antigen Rapid Test in symptomatic patients was 62.16%, which was similar to previous studies with other brands of antigen tests [20, 21]. Since there was only one asymptomatic patient with a positive RT-PCR result, the sensitivity in this group was not calculated in this study. However, previous studies using Standard Q® COVID-19 Rapid Antigen Test performed in Switzerland demonstrated a sensitivity of 28.6% and 41.9% with fair specificity in asymptomatic patients in the ED [9, 21]. The sensitivity of the VTRUST antigen test in the community testing site was not as good as that of a previous antigen study using the INDICAID COVID-19 rapid antigen test in screening centers in Hong Kong, in which the sensitivity and specificity were 84.2% and 99.9%, respectively [10]. Based on the Ct value, the sensitivity of the VTRUST COVID-19 Antigen Rapid Test was 100%, 75%, 27.78%%, and 0% when the Ct value was <20, 20–24.99, 25–30, and >30, respectively. This result shows a similar trend to previous studies and the sensitivity was comparable with the Standard *Q* (Roche, Switzerland) in a study from Germany [22] but slightly inferior to the SARS-CoV-2 Rapid Antigen Test (Roche, Switzerland) in another study from Germany [15]. In a previous systematic review, a total of 48 studies related to rapid antigen testing were included, and the results also revealed that the diagnostic performance of antigen tests varied in different testing kits. The average sensitivity and specificity of SARS-CoV-2 rapid antigen tests were 72% (63.7–79.0%) and 99.5% (98.5–99.8%) for symptomatic patients, as well as 58% (40.2–74.1%) and 98.9% (93.6–99.8%) for asymptomatic patients, respectively [11]. This may be due to different brands of antigen tests and different clinical contexts in each study. The validation for each EUA antigen tests is important to ensure its diagnostic performance in clinical contexts.

According to the collection site, we found that the sensitivity of the VTRUST COVID-19 Antigen Rapid Test was higher in the specimens from the ED (62.50%) than in those from the community testing site (31.82%). High specificity was observed in both the ED and community testing site. There were two differences between the ED and community testing in this study. First, the antigen tests were operated by laboratory staff in our ED and by other clinical personnel in the community testing site. Second, the prevalence was higher in the ED than in the community testing site, and most patients in the ED were symptomatic. A previous study using the Abbott BinaxNOW rapid antigen test revealed a sensitivity of 64.2% with a prevalence of 8.7% in symptomatic patients and a sensitivity of 35.8% with a prevalence of 4.7% in asymptomatic patients [20]. An Italian study using the SD Biosensor STANDARD F COVID-19 Ag FIA test in the ED also showed a sensitivity of 68.0% and 41.9% in symptomatic patients (prevalence of 16.6%) and asymptomatic patients (prevalence of 2.37%), respectively [21]. To clarify the cause of the differences in sensitivity, the sensitivity analysis based on different Ct values was performed in this study. Slightly higher sensitivity with a Ct value of 20–24.99 (ED: 76.47%; community testing site: 66.67%) and lower sensitivity with a Ct value of 25–30 (ED: 25%; community testing site: 33.33%) was found in the ED than in the community testing site. There was no difference in sensitivity when the Ct value was less than 20 or higher than 30. The difference might be mainly attributed to the lower Ct values in the ED (median: 23.55, min-max: 12.88–34.2) than in the community testing site (median: 29.52, min-max: 16.92 to 36.63). Furthermore, these results might imply that the VTRUST COVID-19 Antigen Rapid Test is a suitable tool for point-of-care testing, which could be also performed by other clinical personnel rather than only laboratory-trained staff. However, there were relatively few cases in each Ct value group owing to only 62 positive cases in our study, which may affect the results of the sensitivity analysis. Therefore, our findings should be validated with further research.

There are some limitations to this study. First, it was a retrospective analysis, and the medical information of the screened subjects in the community testing site and caregivers in the ED was unavailable. However, subjects from the community testing site may be regarded as a low-risk population due to the low prevalence rate and caregivers in the ED were a relatively small proportion. Second, the nasopharyngeal swab sample of the subjects for antigen and RT-PCR were not collected at the same time. However, we analyzed the results with paired samples on the same day, and the samples were collected by qualified personnel. The preanalysis factor should have been minimized. Third, there was no quantitative data about the viral load in this study. However, the Ct values of real-time RT-PCR were presented. The previous study revealed that cobas SARS-CoV-2 PCR test on the cobas 6800 system had a limit of detection of 25 to 50 copies/mL, which was also similar to the analytical sensitivity provided by the manufacturer's specification [23, 24]. Finally, the performance of the VTRUST COVID-19 Antigen Rapid Test for detecting the SARS-CoV-2 Omicron variant was not known due to our study period being earlier than the emergence of Omicron variant, which needs to be validated in the further study. However, we investigated different clinical settings corresponding to different prevalence rates in this study. We also performed a sensitivity analysis of the antigen test based on Ct values of real-time RT-PCR to elucidate the possible cause leading to difference results between the ED and community testing site, which provides directions for future research. To the best of our knowledge, this is the first study to evaluate the clinical performance of VTRUST COVID-19 Antigen Rapid Test in the ED and community testing sites.

In conclusion, the VTRUST COVID-19 Antigen Rapid Test showed comparable specificity to real-time RT-PCR both in the ED and community testing site, but the sensitivity was relatively low, especially when the Ct value was higher than 25. An antigen rapid test with adequate specificity is a useful tool to assist in the identification of highly contagious individuals in an epidemic situation. It is crucial to validate the EUA antigen tests in clinical contexts to ensure diagnostic performance in the real world.

## Figures and Tables

**Table 1:** Clinical performance of the VTRUST COVID-19 Antigen Rapid Test with respect to RT-PCR results.

	Overall (95% CI)	ED (95% CI)	Community (95% CI)
Subjects	4022	800	3222
Median *E* gene Ct value (IQR)	24.93 (21.41–28.79)	23.55 (20.31–27.22)	29.52 (24.95–34.59)
Prevalence	1.55% (1.19–1.98%)	5.00% (3.60%–6.75%)	0.69% (0.43%–1.04%)
Sensitivity	51.61% (38.56–64.50%)	62.50% (45.80%–77.27%)	31.82% (13.86%–54.87%)
Specificity	99.44% (99.16–99.65%)	99.61% (98.85%–99.92%)	99.40% (99.07%–99.64%)
PPV	59.26% (47.34–70.18%)	89.29% (72.43%–96.36%)	26.92% (14.72%–44.03%)
NPV	99.24% (99.02–99.41%)	98.06% (97.13%–98.69%)	99.53% (99.37%–99.65%)

RT-PCR, reverse transcription-polymerase chain reaction; ED, emergency department; CI, confidence interval; Ct, cycle threshold; IQR, interquartile range; PPV, positive predictive value; NPV, negative predictive value; COVID-19, coronavirus disease 2019.

**Table 2:** Characteristics and antigen result of patients in the ED.

Characteristic	RT-PCR	
	Positive (*n* = 38)	Negative (*n* = 696)
Age	64 (IQR: 55–72)	64 (IQR: 50–74)
Sex		
Male	22 (57.9%)	410 (58.9%)
Female	16 (42.1%)	286 (41.1%)
Vital signs at triage		
BT (degree Celsius)	36.7 (IQR: 36.4–37.1)	36.6 (IQR: 36.2–37.2)
RR (breaths per minute)	20 (IQR: 18–20)	20 (IQR: 18–20)
SPO_2_ (%)	96 (IQR: 93–97)	98 (IQR: 96–98)
Symptomatic	37 (97.4%)	572 (82.2%)
Fever/Chills	26 (68.4%)	207 (29.7%)
Cough	23 (60.5%)	89 (12.8%)
SOB	18 (47.4%)	107 (15.4%)
Chest pain/tightness	3 (7.9%)	58 (8.3%)
Headache/Dizziness	4 (10.5%)	55 (7.9%)
Fatigue	6 (15.8%)	69 (9.9%)
Loss of taste or smell	2 (5.3%)	2 (0.3%)
Sore throat	7 (18.4%)	22 (3.2%)
Diarrhea	6 (15.8%)	31 (4.5%)
Muscle aches	6 (15.8%)	12 (1.7%)
Abdominal pain	1 (2.6%)	127 (18.2%)
Other symptoms^*∗*^	9 (23.7%)	166 (23.9%)
Asymptomatic	1 (2.6%)	124 (17.8%)
Time from onset to laboratory test (days)		
<7 days	27 (71.1%)	448 (64.4%)
≥7 days	8 (21.1%)	58 (8.3%)
Unknown	2 (5.3%)	66 (9.5%)
Antigen result		
Antigen positive	14 (36.8%)	1 (0.2%)
Antigen negative	24 (63.2%)	695 (99.8%)

^*∗*^Other symptoms included vomiting, poor appetites and drowsy consciousness. RT-PCR, reverse transcription-polymerase chain reaction; ED, emergency department; IQR, interquartile range; BT, body temperature; RR, respiratory rate; SpO2, oxygen saturation; SOB, shortness of breath.

## Data Availability

The data that support the findings of this study are available from the corresponding author upon reasonable request.
